# Bis(2-bromo­pyridinium) hexa­chlorido­stannate(IV)

**DOI:** 10.1107/S160053680800901X

**Published:** 2008-04-10

**Authors:** Basem Fares Ali, Rawhi Al-Far, Salim F. Haddad

**Affiliations:** a Department of Chemistry, Al al-Bayt University, Mafraq, Jordan; b Faculty of Science and IT, Al-Balqa’a Applied University, Salt, Jordan; c Department of Chemistry, The University of Jordan, Amman, Jordan

## Abstract

The asymmetric unit of the title compound, (C_5_H_5_BrN)_2_[SnCl_6_], contains one cation and one half-anion. The [SnCl_6_]^2−^ anion is located on an inversion center and forms a quasi-regular octa­hedral arrangement. Hydrogen-bonding inter­actions of two kinds, *viz*. N—H⋯Cl—Sn and C—H⋯Cl—Sn, along with Cl⋯Br inter­actions [3.4393 (15) Å], connect the ions in the crystal structure into two-dimensional supra­molecular arrays. These supra­molecular arrays are arranged in layers approximately parallel to (110) built up from anions inter­acting with six symmetry-related surrounding cations.

## Related literature

The title salt is isomorphous with the Te-analogue, see: Fernandes *et al.* (2004 ▶). For related literature, see: Al-Far & Ali (2007 ▶); Ali, Al-Far & Al-Sou’od (2007 ▶); Ali & Al-Far (2007 ▶); Ali, Al-Far & Ng (2007 ▶); Allen *et al.* (1987 ▶); Aruta *et al.* (2005 ▶); Awwadi *et al.* (2007 ▶); Bouacida *et al.* (2007 ▶); Ellis & Macdonald (2006 ▶); Hill (1998 ▶); Kagan *et al.* (1999 ▶); Knutson *et al.* (2005 ▶); Li *et al.* (2005 ▶); Mitzi *et al.* (2001 ▶); Raptopoulou *et al.* (2002 ▶); Willett & Haddad (2000 ▶).
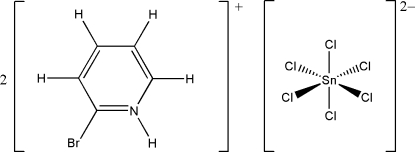


## Experimental

### 

#### Crystal data

(C_5_H_5_BrN)_2_[SnCl_6_]
*M* *_r_* = 649.41
Monoclinic, 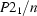

*a* = 9.0843 (14) Å
*b* = 10.6827 (9) Å
*c* = 10.6345 (17) Å
β = 109.843 (11)°
*V* = 970.8 (2) Å^3^
*Z* = 2
Mo *K*α radiation
μ = 6.25 mm^−1^
*T* = 296 (2) K
0.20 × 0.15 × 0.10 mm

#### Data collection

Siemens P4 diffractometer
Absorption correction: ψ scan (*XSCANS*; Siemens, 1996 ▶) *T*_min_ = 0.340, *T*_max_ = 0.535
2385 measured reflections
1791 independent reflections
1343 reflections with *I* > 2σ(*I*)
*R*_int_ = 0.048

#### Refinement

*R*[*F* ^2^ > 2σ(*F* ^2^)] = 0.040
*wR*(*F* ^2^) = 0.097
*S* = 1.05
1791 reflections
98 parameters
H-atom parameters constrained
Δρ_max_ = 0.61 e Å^−3^
Δρ_min_ = −0.73 e Å^−3^

### 

Data collection: *XSCANS* (Siemens, 1996 ▶); cell refinement: *XSCANS*; data reduction: *SHELXTL* (Sheldrick, 2008 ▶); program(s) used to solve structure: *SHELXS97* (Sheldrick, 2008 ▶); program(s) used to refine structure: *SHELXL97* (Sheldrick, 2008 ▶); molecular graphics: *SHELXTL*; software used to prepare material for publication: *SHELXTL*.

## Supplementary Material

Crystal structure: contains datablocks I, global. DOI: 10.1107/S160053680800901X/bh2165sup1.cif

Structure factors: contains datablocks I. DOI: 10.1107/S160053680800901X/bh2165Isup2.hkl

Additional supplementary materials:  crystallographic information; 3D view; checkCIF report

## Figures and Tables

**Table:** 

Sn1—Cl1	2.4216 (13)
Sn1—Cl2	2.4513 (14)
Sn1—Cl3	2.4212 (13)

**Table:** 

Cl1—Sn1—Cl2	89.67 (5)
Cl3—Sn1—Cl1^i^	89.70 (5)
Cl3—Sn1—Cl1	90.30 (5)
Cl3—Sn1—Cl2	90.06 (6)
Cl3—Sn1—Cl2^i^	89.94 (6)

Symmetry code: (i) 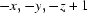.

**Table 2:** Hydrogen-bond geometry (Å, °)

*D*—H⋯*A*	*D*—H	H⋯*A*	*D*⋯*A*	*D*—H⋯*A*
N1—H1⋯Cl2^ii^	0.86	2.45	3.234 (5)	151
C3—H3⋯Cl1^iii^	0.93	2.77	3.646 (6)	158
C5—H5⋯Cl1^iv^	0.93	2.86	3.774 (7)	170

Symmetry codes: (ii) 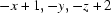; (iii) 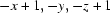; (iv) 
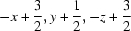
.

**Fig. 1.**
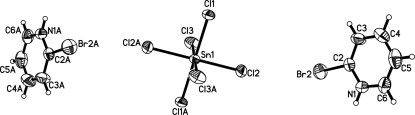
A view of the asymmetric unit of (I), with the atom-numbering scheme. Displacement ellipsoids are drawn at the 50% probability level.

**Fig. 2.**
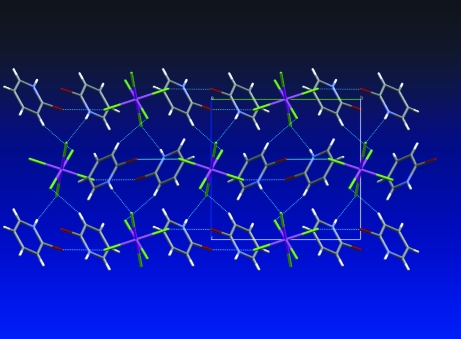
A packing diagram of (I). Hydrogen bonds and Cl···Br interactions are shown as dashed lines.

**Fig. 3.**
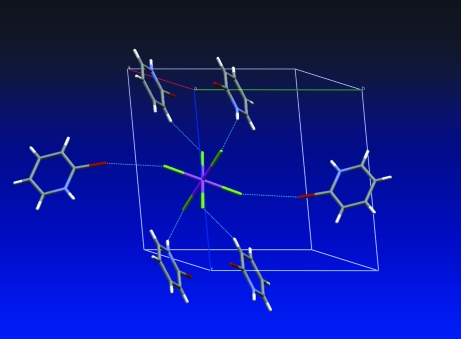
Part of the cell contents of (I), showing Cl···Br and (C,N)—H···Cl intermolecular interactions (dashed lines) for one (SnCl6)2- anion and six surrounding cations.

## supplementary crystallographic information

### Comment

Noncovalent interactions play an important role in organizing structural units in both natural and artificial systems. Hybrid organic-inorganic compounds are of great interest owing to their ionic, electrical, magnetic and optical properties (Hill, 1998; Kagan *et al.*, 1999; Raptopoulou *et al.*, 2002). Tin metal-halo based hybrids are of particular interest as being materials with interesting optical and magnetic properties (Aruta *et al.*, 2005; Knutson *et al.*, 2005; Mitzi *et al.*, 2001; Kagan *et al.*, 1999). We are currently carrying out studies about lattice including different types of intermolecular interactions. Our strategy is to use aromatic compounds to encourage aryl···aryl packing arrangements of various types, using substituted pyridinium in order to facilitates associations, and halo salts that can involve in *X*···*X* interactions as well as *X*···aryl and *X*···H interactions. Within our research of hybrid compounds containing tin metal (Al-Far & Ali 2007; Ali, Al-Far & Al-Sou'od, 2007; Ali & Al-Far, 2007; Ali, Al-Far & Ng, 2007), the crystal structure of the title salt, (I), has been investigated.

The asymmetric unit of (I) contains one cation and one-half anion (Fig. 1). The (SnCl_6_)^2-^ anion lies on an inversion center, in a quasi-octahedral geometry (Table 1). The Sn—Cl bond lengths are almost invariant, but Sn—Cl2 is longer than the others (involved in the shortest hydrogen bonds). These lengths fall within the range of tin-chloride distances reported previously for compounds containing (SnCl_6_)^2-^ anions (Bouacida *et al.*, 2007; Ellis & Macdonald, 2006; Li *et al.*, 2005; Willett & Haddad, 2000). Bond lengths and angles within the cation are as expected (Allen *et al.*, 1987).

The packing of the structure (Fig. 2) can be described as layers of alternating anions (zigzag orientation) along the face parallel to *b*-axis and diagonal to *ac* plane. Each (SnCl_6_)^2-^ anion is surrounded by six cations *via* four equatorial (C,*N*)—H···Cl interactions (Table 2) and two axial Cl···Br interactions [Cl3···Br2*^i^* = 3.4393 (15) Å; symmetry code: (*i*) -1/2 + *x*, -1/2 - *y*, -1/2 + *z*; Fig. 3). This arrangement of molecules appears as layers approximately parallel to [110]. It is noteworthy that structural and theoretical results (Awwadi *et al.*, 2007; and references therein), show the significance of linear C—Y···*X*^-^ (in this case C—Cl···Br) synthons in influencing structures of crystalline materials and in use as potential building blocks in crystal engineering *via* supramolecular synthesis.

The intermolecular hydrogen bonds (Table 2) and Cl···Br interactions would therefore add some lattice stability. This is evident in the isostructurality with the reported Te analogue (Fernandes *et al.*, 2004).

### Experimental

Warm solution of SnCl_4_ (1.0 mmol) dissolved in absolute ethanol (10 ml) and concentrated HCl (1 ml), was added dropwise to a stirred hot solution of 2-bromopyridine (1 mmol) dissolved in ethanol (10 ml). The mixture was treated with another 2 ml of concentrated HCl and refluxed for 2 h, then cooled, filtered off, and allowed to stand undisturbed at room temperature. The salt crystallized over 1 d as nice yellow block crystals (yield: 89.6%).

### Refinement

H atoms were positioned geometrically, with N—H = 0.86 Å (for NH) and C—H = 0.93 Å for aromatic H, and constrained to ride on their parent atoms, with *U*_iso_(H) = 1.2*U*_eq_(C, N).

### Crystal data

**Table:**

(C_5_H_5_BrN)_2_[SnCl_6_]	*F*_000_ = 612
*M**_r_* = 649.41	*D*_x_ = 2.222 Mg m^−^^3^
Monoclinic, *P*2_1_/*n*	Mo *K*α radiation λ = 0.71073 Å
Hall symbol: -P 2yn	Cell parameters from 90 reflections
*a* = 9.0843 (14) Å	θ = 1.6–27.4º
*b* = 10.6827 (9) Å	µ = 6.25 mm^−^^1^
*c* = 10.6345 (17) Å	*T* = 296 (2) K
β = 109.843 (11)º	Block, yellow
*V* = 970.8 (2) Å^3^	0.20 × 0.15 × 0.10 mm
*Z* = 2	

### Data collection

**Table:**

Siemens P4 diffractometer	1791 independent reflections
Radiation source: fine-focus sealed tube	1343 reflections with *I* > 2σ(*I*)
Monochromator: graphite	*R*_int_ = 0.048
Detector resolution: 3 pixels mm^-1^	θ_max_ = 25.5º
*T* = 296(2) K	θ_min_ = 2.8º
ω scans	*h* = −1→11
Absorption correction: ψ scan(XSCANS; Siemens, 1996)	*k* = −12→1
*T*_min_ = 0.340, *T*_max_ = 0.535	*l* = −12→12
2385 measured reflections	

### Refinement

**Table:**

Refinement on *F*^2^	Hydrogen site location: inferred from neighbouring sites
Least-squares matrix: full	H-atom parameters constrained
*R*[*F*^2^ > 2σ(*F*^2^)] = 0.040	*w* = 1/[σ^2^(*F*_o_^2^) + (0.0417*P*)^2^ + 0.8983*P*] where *P* = (*F*_o_^2^ + 2*F*_c_^2^)/3
*wR*(*F*^2^) = 0.097	(Δ/σ)_max_ < 0.001
*S* = 1.05	Δρ_max_ = 0.61 e Å^−^^3^
1791 reflections	Δρ_min_ = −0.73 e Å^−^^3^
98 parameters	Extinction correction: SHELXL97 (Sheldrick, 2008), Fc^*^=kFc[1+0.001xFc^2^λ^3^/sin(2θ)]^-1/4^
Primary atom site location: structure-invariant direct methods	Extinction coefficient: 0.0145 (10)
Secondary atom site location: difference Fourier map	

### Fractional atomic coordinates and isotropic or equivalent isotropic displacement parameters (Å^2^)

**Table:**

	*x*	*y*	*z*	*U*_iso_*/*U*_eq_	
Sn1	0.0000	0.0000	0.5000	0.0271 (2)	
Br2	0.68293 (9)	0.00098 (6)	0.92745 (7)	0.0595 (3)	
Cl1	0.15178 (16)	0.07315 (13)	0.36630 (13)	0.0376 (4)	
Cl2	0.24265 (16)	−0.03276 (15)	0.68883 (14)	0.0474 (4)	
Cl3	0.01440 (19)	−0.21235 (13)	0.42546 (16)	0.0500 (4)	
N1	0.8532 (5)	0.1873 (5)	1.0891 (4)	0.0410 (11)	
H1	0.7983	0.1643	1.1369	0.049*	
C6	0.9552 (7)	0.2804 (5)	1.1327 (6)	0.0485 (15)	
H6	0.9655	0.3203	1.2130	0.058*	
C5	1.0445 (8)	0.3170 (7)	1.0591 (7)	0.0612 (19)	
H5	1.1162	0.3820	1.0881	0.073*	
C2	0.8330 (6)	0.1285 (5)	0.9736 (5)	0.0368 (12)	
C4	1.0264 (8)	0.2556 (7)	0.9409 (7)	0.0621 (19)	
H4	1.0880	0.2782	0.8904	0.075*	
C3	0.9181 (7)	0.1612 (6)	0.8968 (6)	0.0518 (16)	
H3	0.9039	0.1210	0.8160	0.062*	

### Atomic displacement parameters (Å^2^)

**Table:**

	*U*^11^	*U*^22^	*U*^33^	*U*^12^	*U*^13^	*U*^23^
Sn1	0.0278 (3)	0.0278 (3)	0.0285 (3)	0.0023 (2)	0.0134 (2)	0.0006 (2)
Br2	0.0574 (5)	0.0572 (4)	0.0634 (5)	−0.0230 (3)	0.0197 (3)	−0.0074 (3)
Cl1	0.0379 (7)	0.0438 (7)	0.0379 (7)	−0.0022 (6)	0.0216 (6)	0.0021 (6)
Cl2	0.0334 (7)	0.0680 (9)	0.0382 (8)	−0.0004 (7)	0.0088 (6)	0.0147 (7)
Cl3	0.0604 (10)	0.0319 (7)	0.0698 (10)	0.0009 (7)	0.0378 (8)	−0.0086 (7)
N1	0.040 (3)	0.050 (3)	0.035 (2)	−0.003 (2)	0.016 (2)	0.001 (2)
C6	0.042 (3)	0.045 (3)	0.051 (4)	0.000 (3)	0.005 (3)	−0.010 (3)
C5	0.046 (4)	0.050 (4)	0.076 (5)	−0.010 (3)	0.005 (3)	0.007 (4)
C2	0.031 (3)	0.036 (3)	0.041 (3)	−0.002 (2)	0.009 (2)	0.001 (2)
C4	0.050 (4)	0.076 (5)	0.069 (5)	−0.013 (4)	0.033 (3)	0.002 (4)
C3	0.054 (4)	0.062 (4)	0.050 (4)	−0.009 (3)	0.031 (3)	−0.011 (3)

### Geometric parameters (Å, °)

**Table:**

Sn1—Cl1	2.4216 (13)	N1—H1	0.8600
Sn1—Cl2	2.4513 (14)	C6—C5	1.362 (9)
Sn1—Cl3	2.4212 (13)	C6—H6	0.9300
Sn1—Cl1^i^	2.4216 (13)	C5—C4	1.378 (10)
Sn1—Cl2^i^	2.4513 (14)	C5—H5	0.9300
Sn1—Cl3^i^	2.4212 (13)	C2—C3	1.347 (8)
Br2—C2	1.871 (5)	C4—C3	1.375 (9)
N1—C6	1.331 (8)	C4—H4	0.9300
N1—C2	1.336 (7)	C3—H3	0.9300
			
Cl1—Sn1—Cl2	89.67 (5)	C2—N1—H1	118.9
Cl3—Sn1—Cl1^i^	89.70 (5)	N1—C6—C5	119.6 (6)
Cl3—Sn1—Cl1	90.30 (5)	N1—C6—H6	120.2
Cl3—Sn1—Cl2	90.06 (6)	C5—C6—H6	120.2
Cl3—Sn1—Cl2^i^	89.94 (6)	C6—C5—C4	118.6 (6)
Cl1—Sn1—Cl2^i^	90.33 (5)	C6—C5—H5	120.7
Cl3^i^—Sn1—Cl3	180.0	C4—C5—H5	120.7
Cl3^i^—Sn1—Cl1^i^	90.30 (5)	N1—C2—C3	120.5 (5)
Cl3^i^—Sn1—Cl1	89.70 (5)	N1—C2—Br2	116.4 (4)
Cl1^i^—Sn1—Cl1	180.0	C3—C2—Br2	123.1 (5)
Cl3^i^—Sn1—Cl2^i^	90.06 (6)	C3—C4—C5	120.7 (7)
Cl1^i^—Sn1—Cl2^i^	89.67 (5)	C3—C4—H4	119.6
Cl3^i^—Sn1—Cl2	89.94 (6)	C5—C4—H4	119.6
Cl1^i^—Sn1—Cl2	90.33 (5)	C2—C3—C4	118.4 (6)
Cl2^i^—Sn1—Cl2	180.0	C2—C3—H3	120.8
C6—N1—C2	122.3 (5)	C4—C3—H3	120.8
C6—N1—H1	118.9		
			
C2—N1—C6—C5	−0.9 (9)	C6—C5—C4—C3	1.4 (11)
N1—C6—C5—C4	−0.1 (10)	N1—C2—C3—C4	0.6 (9)
C6—N1—C2—C3	0.7 (9)	Br2—C2—C3—C4	−179.4 (5)
C6—N1—C2—Br2	−179.4 (4)	C5—C4—C3—C2	−1.6 (11)

Symmetry codes: (i) −*x*, −*y*, −*z*+1.

### Hydrogen-bond geometry (Å, °)

**Table:**

*D*—H···*A*	*D*—H	H···*A*	*D*···*A*	*D*—H···*A*
N1—H1···Cl2^ii^	0.86	2.45	3.234 (5)	151
C3—H3···Cl1^iii^	0.93	2.77	3.646 (6)	158
C5—H5···Cl1^iv^	0.93	2.86	3.774 (7)	170

Symmetry codes: (ii) −*x*+1, −*y*, −*z*+2; (iii) −*x*+1, −*y*, −*z*+1; (iv) −*x*+3/2, *y*+1/2, −*z*+3/2.
